# *Haemophilus Aphrophilus* Associated Spleen Abscess: An Unusual Presentation of Subacute Endocarditis

**DOI:** 10.4021/jocmr803w

**Published:** 2012-05-15

**Authors:** Ya-Chih Tien, Chia-Chu Chang, Yuan-Meng Liu

**Affiliations:** a Department of Internal Medicine, Changhua Christian Hospital, Changhua City, Taiwan

**Keywords:** Endocarditis, HACEK, *Haemophilus aphrophilus*, Spleen abscess

## Abstract

The HACEK group of bacteria (*Haemophilus* spp., *Actinobacillus actinomycetemcomitans, Cardiobacterium hominis, Eikenella corrodens*, *Kingella* spp.), is uncommon pathogens of infective endocarditis, but can cause life-threatening events such as heart failure or formation of lethal emboli. Here we report a 58-year-old Asian man with a past history of congenital valvular heart disease who presented with sudden onset of left flank pain followed by fever with chills for 2 weeks. Abdominal computed tomography (CT) indicated a 1.6 cm abscess in the spleen. Culturing indicated the presence of *Haemophilus aphrophilus*. We diagnosed the patient with subacute endocarditis complicated with spleen abscess. The patient recovered fully after two weeks antibiotic (Ceftriaxone) treatment. Clinicians should give further attention to infective endocarditis caused by bacteria in the HACEK group in patients with metastatic infection such as spleen abscess with suspected valvular heart disease.

## Introduction

Endocarditis due to *Haemophilus * spp. is rare, and only 0.8 - 1.3% cases of adult endocarditis are caused by these bacteria [1]. *Haemophilus * spp. belongs to HACEK group of Gram-negative bacteria, species that together are responsible for 3 - 8% of all cases of infective endocarditis. Patients who have HACEK-associated endocarditis almost always present with subacute endocarditis. The symptoms of subacute endocarditis often develop insidiously over many weeks, and fatal complications, such as heart failure or formation of emboli, may occur [2]. Here we report a case of *Haemophilus aphrophilus* infective endocarditis and review the literature on infections caused by HACEK organisms, with a focus on *Haemophilus aphrophilus*-associated infections.

## Case Report

A 58-year-old man was admitted to our hospital with sudden onset of left flank muscle strain with flank pain by moving sofa at home, followed by fever with chills for two weeks. He has a history of congenital valvular heart disease but no symptoms of heart failure. He was admitted to our hospital due to left flank pain and mild fever with chills (two episodes per day) that persisted despite treatment with analgesics and antifebrile drugs.

On admission, his blood pressure was 124/68 mmHg, body temperature was 38 ºC, heart rate was 118 beats per minute, and respiratory rate was 18 breaths per minute without distress. His heart had a regular beat with systolic murmur (Grade 3) that was overt at the right upper side sternum and apex. A left flank knocking pain with no evident skin lesion was also recorded. His Chest-X-ray indicated clear lung field but borderline cardiomegaly, suggested left ventricle hypertrophy (LVH). Laboratory studies measured white blood cell (WBC) count (11,100/µL (neutrophil-segment 84.3%)), hemoglobin (10.1 g/dL (MCV, 76.5 fL)), platelet count (287,000/µL), creatinine (1.12 mg/dL), Na (133 mmol/L), K (4.7 mmol/L), C-reactive protein (CRP) (13.16 mg/dL), and erythrocyte sedimentation rate (ESR) (114 mm/h). The patient described the left flank pain as persistent, dull, and aggravated by changing of position. He denied any other discomfort, such as malaise, loss of body weight, dyspnea on exertion, chest discomfort, nausea, diarrhea, or dysuria. He also denied any history of travel, trauma, intravenous drug use, or recent dental care.

**Figure 1:**
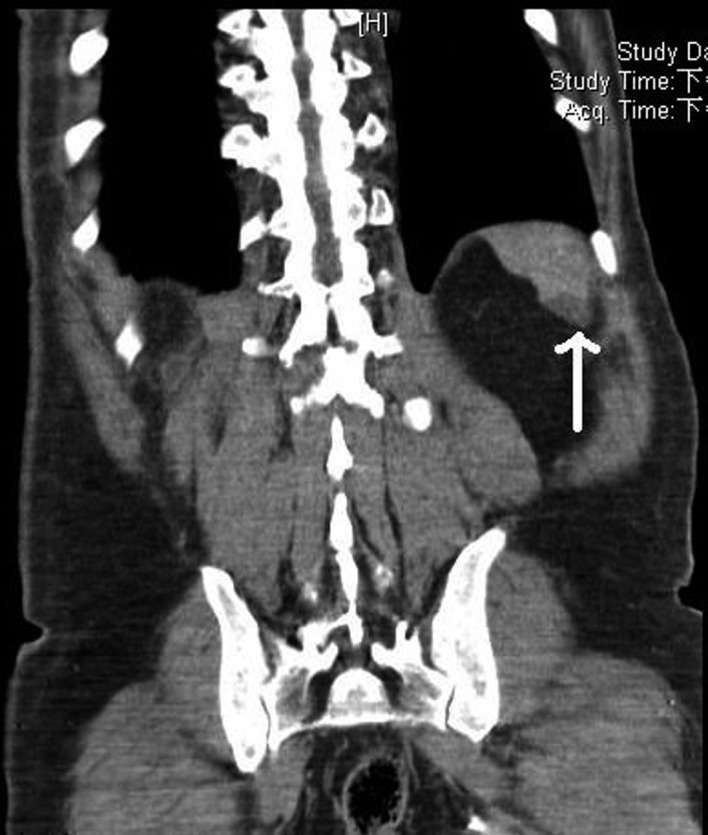
Abdominal CT indicating a nodular lesion at the spleen (point by white arrow) that was 1.6 cm in diameter and showed mild enhancement in a post-contrast study, indicative of a spleen abscess or infarction.

Abdominal computed tomography (CT) was performed on suspicion that the fever and left flank pain were related to an abdominal infection. This CT indicated a 1.6 cm nodule lesion in the spleen (Fig. 1), compatible with site of tenderness, suggestive of spleen abscess or infarction. Empiric therapy with ceftriaxone 2 g every 12 hour was prescribed initially.

Echocardiography was arranged under consideration of valvular heart disease and unexplained metastatic infection of spleen emboli, which indicated preserved left ventricular (LV) systolic function (ejection fraction, 62%), severe aortic regurgitation (AR) (pressure gradient, 60 mmHg), and a flail mitral valve (MV) with severe mitral regurgitation (MR) (PG, 160 mmHg). Echocardiography indicated no evidence of infective vegetation. Four days after admission, two independent blood cultures yielded *Haemophilus aphrophilus*.

The findings described above fulfill the Duke criteria for infective endocarditis (one major finding: positive blood culture; three minor findings: predisposition for heart disease, fever, and vascular emboli). Thus, we diagnosed our patients with *Haemophilus aphrophilus* associated subacute endocarditis, with a predisposition for congenital valvular heart disease, complicated with spleen abscess which thought to be associated with hemogenous seeding by hematoma caused by flank muscle strain injury. We kept Ceftriaxone (2000 mg/12 h) by intravenous drip. The patient’s fever gradually subsided and his left flank pain completely resolved within two weeks. We maintained Ceftriaxone use for a total of two weeks, at which time the patient’s WBC count was 6200/µL (neutrophil-segment, 63.8%), CRP was 0.15 mg/dL, ESR was 38 mm/h, and a liver echogram indicated no evidence of spleen abscess. The patient denied any episode of fever or left flank pain during entire course of treatment, we classified the patient as free from disease.

## Discussion

Infective endocarditis, which is characterized by the presence of a lesion (vegetation) consisting of platelets, fibrin, microorganisms, and inflammatory cells in the endocardium [3], was first described in English by William Osler in 1885. The Duke criteria are the most widely used standard for diagnosis of infective endocarditis [4]. In the United States and Western Europe, the incidence of community-acquired native valve endocarditis is about 1.7 to 6.2 cases per 100,000 person-years and the most common infective pathogen is *Staphylococcus aureus*, followed by *Streptococcus* spp. [3].

The HACEK group of Gram-negative bacilli is considered uncommon pathogens of infective endocarditis, and account for only about 3 - 8% of all cases [5]. Bacteria in the HACEK group are characterized by: (1) their presence in the normal flora of oral and upper airways; (2) low virulence; (3) increased presence in patients with recent dental care, history of heart valve abnormalities, or with a prosthetic heart valves [2]; and (4) slow growth in culture, requiring prolonged incubation time, always more than 48 h (mean of 3.3 days and occasionally as long as 7 days), typically resulting in culture-negative results. Thus, most cases of infective endocarditis (IE) caused by HACEK organisms are initially considered subacute [6]. However, patients with subacute endocarditis caused by HACEK organisms tend to develop symptoms over many weeks, and may eventually harbor life-threatening large vegetations that lead to emboli or heart failure due to the delay in diagnosis [7].

*Haemophilus* spp., including *H. parainfluenzae*, *H. aphrophilus*, and *H. paraphrophilus*, are responsible for a large proportion of HACEK-associated endocarditis (43%) [6]. Goldberg and Katz reported pre-existing valvular abnormalities in 50% of cases of *Haemophilus* endocarditis, and a mortality rate as high as 35%, with half of all mortalities due to septic emboli [6]. Clemence et al. [1] reviewed 42 cases of *Haemophilus* endocarditis in France from 1980 to 1995. They reported that 15 cases (35.7%) presented with arterial emboli when diagnosis was confirmed, whereas a review of pre-1980 data indicated that 29 of 40 cases (72.5%) had arterial emboli. In all of the 42 cases reviewed by Clemence et al. [1], there were only two deaths due to endocarditis (4.8%). They also mentioned that the mean duration before diagnosis was 34 days (range, 2 - 330 days), thus underlining the fact that HACEK related endocarditis is difficult to diagnose.

Huang ST et al. [8] searched Medline for publications on *Haemophilus aphrophilus* related infection from 1990 to 2003 and identified 28 cases. The mean age was 47.4 years (range: 7 - 73 years) and 21 cases (75%) were male. The major manifestations were bone and joint infections (9 cases, 32%) and infective endocarditis (7 cases, 25%); other sites included ophthalmic infections (3 cases, 10%), meningitis, brain abscess, cervical lymphadenitis, facial cellulitis, empyema, and purulent pericarditis, and tamponade. Among these 28 patients, 11 patients (39%) reported recent dental procedures.

We searched the infectious disease database in our hospital (Changhua Christian Hospital, Changhua City, Taiwan) from January 2005 to December 2010 and identified 11 cases in which culturing yielded *Haemophilus aphrophilus*. Five of these patients had *H. aphrophilus* bacteremia. Three patients had confirmed diagnosis of *H. aphrophilus* endocarditis (including the case reported here) and the other two died during hospitalization due to sudden cardiac arrest without definite diagnosis, but possible due to initially subacute endocarditis. The other cultures of *H. aphrophilus* were isolated from a neck abscess (1 pt), nose tissue (2 pts), thigh necrotizing fasciitis (1 pt), endometrium (1 pt), and sputum (1 pt).

In conclusion, the prevalence of HACEK-mediated infective endocarditis is low, but slowly growing HACEK colonies may nonetheless cause life-threatening heart failure or emboli. We suggest that further attention be given to HACEK-mediated infective endocarditis, especially in patients with known valvular heart disease who presented with metastatic infection (ex: spleen abscess) so that treatment can be initiated as soon as possible. We also suggest two weeks of intravenous ceftriaxone injection may be an effective therapy for such kind of infection.
